# A Closed Parameterization of DNA–Damage by Charged Particles, as a Function of Energy — A Geometrical Approach

**DOI:** 10.1371/journal.pone.0110333

**Published:** 2014-10-23

**Authors:** Frank Van den Heuvel

**Affiliations:** 1 CRUK/MRC Oxford Institute for Radiation Oncology, Department of Oncology, University of Oxford, Oxford, United Kingdom; 2 Laboratory for experimental radiotherapy, Department of Oncology, University of Leuven, Leuven, Belgium; University of California Davis, United States of America

## Abstract

**Purpose:**

To present a closed formalism calculating charged particle radiation damage induced in DNA. The formalism is valid for all types of charged particles and due to its closed nature is suited to provide fast conversion of dose to DNA-damage.

**Methods:**

The induction of double strand breaks in DNA–strings residing in irradiated cells is quantified using a single particle model. This leads to a proposal to use the cumulative Cauchy distribution to express the mix of high and low LET type damage probability generated by a single particle. A microscopic phenomenological Monte Carlo code is used to fit the parameters of the model as a function of kinetic energy related to the damage to a DNA molecule embedded in a cell. The model is applied for four particles: electrons, protons, alpha–particles, and carbon ions. A geometric interpretation of this observation using the impact ionization mean free path as a quantifier, allows extension of the model to very low energies.

**Results:**

The mathematical expression describes the model adequately using a chi–square test (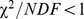). This applies to all particle types with an almost perfect fit for protons, while the other particles seem to result in some discrepancies at very low energies. The implementation calculating a strict version of the RBE based on complex damage alone is corroborated by experimental data from the measured RBE. The geometric interpretation generates a unique dimensionless parameter 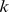 for each type of charged particle. In addition, it predicts a distribution of DNA damage which is different from the current models.

## Introduction

The biological effect of ionizing radiation on human cells is believed to be related to the generation of damage in the DNA–molecule located in the cell's nucleus [1]. The physical mechanism is the ionization of the DNA macro molecule, generating lesions in the molecular structure, either by direct ionization or by the generation of radicals in the vicinity of the DNA which then indirectly damage it. These events (direct or indirect) can create several types of damage to the DNA by combining a number of lesions into a cluster, which can only happen if they occur in close proximity (typically within one turn of the DNA–helix). The most prevalent of these damage types are base damage (2 lesions), followed by single strand breaks (SSB) (3 lesions), double strand breaks (DSB) (4 lesions), and locally multiple damage sites (LMDS). The latter are clusters of different types of damage occurring close to each other. It is shown that base as well as SSB damage is not likely to be a deciding factor in the destruction of cells, due to the efficient repair mechanisms which exist in the cell [2]. The combination of double strand breaks and LMDS's is likely to be the root cause for cell kill [3].

To quantify the amount of ionizing interactions in a medium, the physical notion of dose can be used. Dose is defined as the amount of energy deposited in a medium per unit mass and is expressed in Joule(J) per kg or Gray (Gy). In the case of dose deposition by charged particles the Bethe–formalism is used. This describes ionization events in a medium in terms of energy loss of the charged particles in inelastic collisions with the electrons of the medium, through the notion of mass stopping power (*dE*/*ρdx*). In his seminal work already in 1930, Bethe showed that there is an intimate relationship between stopping power on the one hand, and energy (i.e. speed), charge, and the medium in which the interaction takes place on the other hand [4]. A further extension taking into account the possibility of the charged particle picking up electrons, thereby changing the stopping power was introduced by Barkas [5], using the concept of an effective charge.

In radiation biology, linear energy transfer (LET) is used rather than stopping power. LET is identical to stopping power with the energy delivered to *δ*–rays (i.e. highly energetic knock on electrons) subtracted. This quantity is called restricted stopping power. As such, LET is a measure for the density of ionization taking place along the track of a charged particle through a medium. Due to its close relationship with stopping power, it follows that there is a close relationship between LET and the kinetic energy of the depositing particle. From observation a dearth of DSB's and LMDS's was shown to be related to low LET irradiations, while an increased number of both for the same dose is seen high in hight LET irradiations [1]. Brenner and Ward [6] argued that DSB and LMDS damage was related to multiple interactions by single particles, rather than the combination of single strand breaks generated by single particles. In the field of microdosimetry, this is taken a step further by defining the notion of lineal energy which introduces the amount of energy deposited along lines confined in a convex geometric shape with a given distribution of cord lengths estimating the energy deposited in various shapes, which can be used for measurement (i.e. spheres, cylinders).

Extending this, it is natural to propose a model where distance between ionizations along these lines plays a significant role in the generation of DNA–damage. A full listing and treatment of these quantities can be found in the ICRU reports 16, 19, and 36 [7]–[9].

To describe the damage impact of charged particles on the DNA–structure, the science community has taken its recourse to using Monte Carlo simulations to quantify the damage introduced [10], [11]. A more fundamental analytical approach is currently lacking, due to the underlying complexity of the DNA molecule, and the paucity of the available experimental data. The data which is available is mainly provided in terms of relative biological effective dose (RBE), a quantity combining physical, spectral, chemical, and biological factors, all of which hamper ab–initio calculations.

Monte Carlo calculations are able to predict the induction of simple or complex damage as well as induction of single and double strand breaks in DNA–molecules. These findings are interpreted using the Bethe–Barkas formalism in terms of LET and show that high LET particles indeed introduce more complex damage.

In this paper we develop a parameterization using a simple geometrical model, that describes the behavior as calculated by the Monte Carlo codes. We also show that this formalism describes the current knowledge well.

## Methods and Materials

### Theory

We use the single charged particle model as proposed by Brenner and Ward, distinguishing three types of interaction results: Low LET, high LET, and intermediate LET mode. The specifics of each mode are explained below.

**Figure 1:**
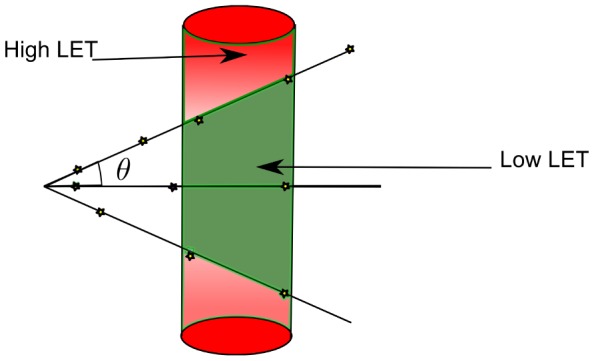
A schematic model of a source of charged particles with a given mean free path length (i.e. a given energy), which is comparable with the diameter of the sensitive cell volume. As the angle (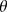) of the particle's path with respect to the normal to the axis of the structure increases the chance that more than a single event will occur in the volume. This implies that if the angle (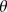) is larger than the one for which the projection of the average length between interactions equals the diameter, more high LET events will be registered.

1. Low LET: A single particle is generally not able to generate lesions close enough together to induce double strand breaks at each interaction. It is clear that DSB's can be generated but in a limited fashion and that we use the word lesion in liberal fashion to indicate an interactive event which has damage as a consequence.
2. High LET: The particle has the possibility to generate multiple lesions irrespective of any geometrical considerations. We implicitly assume that the double strand break damage is the result of multiple interactions by one particle. How exactly this damage is introduced (direct or indirect) is outside the scope of this article. An implicit assumption however is that ionizing events need to be geometrically close to the DNA structure.
3. Intermediate: In given geometric circumstances it is possible for the charged particle to generate DSB–damage, in a high–LET manner, depending on the angle under which the particle hits the sensitive volume (Fig. 1).

As a surrogate to categorize the charged particle in one of the types defined above, we use the mean path length between ionizing interactions in a medium consistent with the atomic make up of a DNA–molecule for the type of particle under consideration. In the remainder, we denote this with 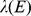, where 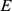 is the kinetic energy of the particle. If 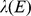 is large relative to the sensitive volume, then the lesions on average are too far apart and only damage types related to a few lesions can occur (i.e. SSB and base damage). Charged particles with such energies will be part of the first category. If on the other hand 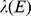 is small then the probability of lesions creating more complex clusters of damage close together will be higher. Charged particles with this property will be in the high LET category. Finally, charged particles with intermediate distances between ionizing events have the capability of generating DSB and LMDS damage depending on other factors than 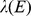 alone. In this model we use the geometric direction of the path of the charged particle as a parameter. In Figure 1 a schematic model of this approach is shown. This implies that only a limited amount of directions are available to contribute to the amount of complex damage in the manner as outlined for the high–LET type interactions. This happens when for a particle of a given energy the quantity 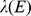 is slightly larger than the maximal distance between two DNA–damage lesions to be considered as being in the same cluster (usually about 10 base pairs (bp)). Due to the finite thickness of the sensitive volume it is possible to behave in a high–LET fashion depending on the angle with which the particle's path crosses the volume. This occurs when the projection of the path is smaller than the previously determined maximum.

### Equivalence Principle

In the case of irradiation with charged particles all directions of the particle's paths are possible as are all rotational positions of the DNA–structure. A particle that interacts (i.e. that creates a lesion) at the surface of a given sensitive volume has limited possibilities to interact again given that on average, a specific distance (which depends on the particle energy) has to be travelled before it interacts again. The next interaction's position is then limited by the constraints outlined above if it is to fall within the sensitive volume. This first interaction can happen anywhere along the volume, but the constraints are relative to the position of that point. This implies that we can invoke an equivalence principle and reduce the problem to that of an isotropic point source positioned at the surface of a sensitive volume.

### Mathematical expression of the equivalence principle

We need to calculate what fraction of the paths starting in the given point can interact with the sensitive volume given the fact that there is a length within which this is not likely, provided by 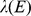, and that there is a maximal distance (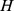) that disqualifies the generated lesion to be registered in the same cluster. We have reduced this problem to that of the distribution of projections of a point source on a line–piece, the solution to which is known as the Cauchy–distribution [12], and is described by the Lorenz function 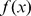 with 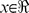 expressed as follows:
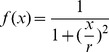
(1)


**Figure 2:**
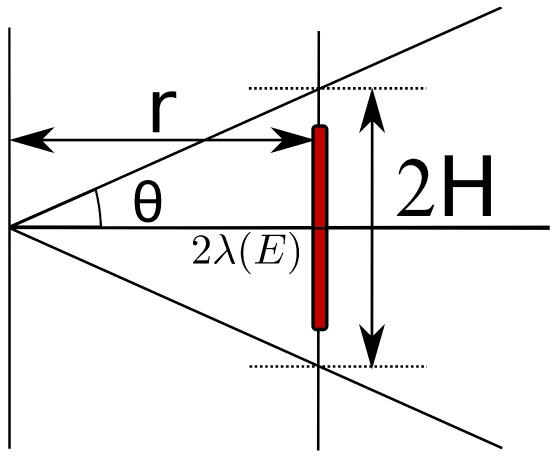
The abstracted version of Figure 11 describes the distribution of horizontal distances at which a line segment tilted at a random angle 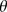 cuts the x–axis. Only particles with large angles contribute to double strand break events by combining damage generated by a single particle. The red line indicates the “forbidden” area as (on average) this distance between the ionization events is observed.

From Figure 2, it follows that the contribution 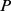 for a given energy of charged particles to high LET events is proportional to:
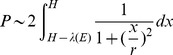
(2)


Performing the calculation, we obtain:
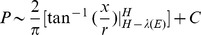
(3)




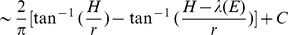
(4)

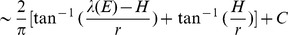
(5)


This implies that the amount of DSB–damage for a given dose and given energy of the charged particle is governed by the following expression.
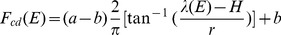
(6)


The change from a low to a high LET regimen occurs over a small energy interval. In such a small interval the average distance dependence on the energy of the particle can be approximated with a linear function. Therefore, we expect the energy dependence of the contribution of complex damage to follow the same form as in Equation 6 yielding the following expression, with H = 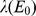 and r = 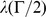, 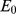 being the energy, where the change in DSB is maximized and 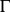 a measure for the width of the slope (i.e. the full width at half maximum in differential energy space).
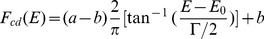
(7)


With the parameters 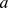 and 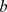 related to the levels 1 and 2 as outlined above. From boundary conditions we find that at very large energies (i.e. 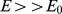) the expression is reduced to minimal number of double interactions (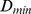) which is equal to 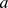. The value of 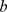 is related to the maximal number of double interactions (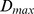) as follows:
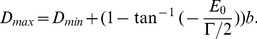
(8)


The formalism using energy alone allows us to forego specific assumptions regarding the dimensions of the DNA–molecule. Furthermore, it also allows us to apply this technique to particles where the values for 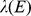 are less well known. In addition, it allows us to test this formalism using experimentally available data which is available as a function of energy.

### Validation using Monte Carlo Simulations

The use of microdosimetric calculations has provided important insight into the mechanisms and effects of radiation deposition. In the past, Monte Carlo simulations of charged particle deposition by various modalities were used to quantify and typify the kinds of damage introduced by the different modalities [10].

The Monte Carlo Damage Simulation code (MCDS) developed by Semenenko and Stewart, generates spatial maps of the damaged nucleotides forming many types of clustered DNA lesion, including single-strand breaks (SSB), double strand breaks (DSB), and individual or clustered base damages [11]. This approach has been shown to yield a linear relationship of the number of generated DSB's up to a high dosage. It follows that this parameterization also provides the possibility to link dose to damage. In this paper, MCDS version 3.0 was used with the parameters described below. The DNA length which was chosen to be 1 Gbp (Giga base pairs) and a nucleus diameter of 5* µ*m. In the MCDS software, the geometry of the DNA–molecule is not an explicit parameter. Here four parameters are used: 1) the DNA–segment length 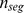, which is an *ad hoc* parameter expressed as base pairs 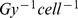, 2) the number of strand breaks generated 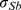, 3) the number of base pair damages generated 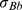 by defining 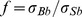, and 4) a parameter 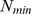 (bp) describing the minimal separation for damage to be apart not to be counted as being in the same cluster. The values of these parameters is determined on the basis of other simulations and measurements. For a more in–depth treatment of these parameters we refer to the work by Semenenko and Stewart [13]. Variable input parameters MCDS were; the modality (i.e. energy depositing particle (electron, proton,…)), the energy (in MeV), and the oxygen concentration in %. In the implementation described here we chose to omit any oxygen enhancement as this could be a confounding factor and is the subject of another study. In this study it was found that oxygen only changed the amount of damage in the low LET regimen, leaving the formalism unchanged (data not shown). Therefore, a concentration of 0% oxygen was used. For every particle type at the relevant kinetic energies, all complex damage was noted per Gy, per cell and per kinetic energy.

### Fitting procedure

The ultimate goal was to fit the complex damage function to the data obtained by the Monte Carlo simulation. The parameters that need fitting are the energy position (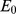) the width of the underlying Cauchy distribution (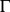) and the parameters 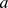 and 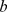. If a regular fit (i.e. all parameters fit at the same time) is performed we see strong co–variances between the parameters. To come to meaningful results we opted to perform a two step procedure: First, we eliminate the parameters 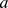 and 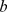 by fitting the differential, thereby reducing expression 6 to the Lorenz function.
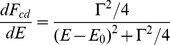
(9)


This is also mathematically equivalent to the fit of a Breit–Wigner resonance in high energy physics [14]. In a second fit–procedure, the remaining variables 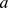 and 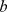 are fit using the cumulative Cauchy function. The fitting procedures were performed in the gnuplot–software using a Levenberg–Marquardt minimization routine (http://www.gnuplot.info).

## Results

**Figure 3:**
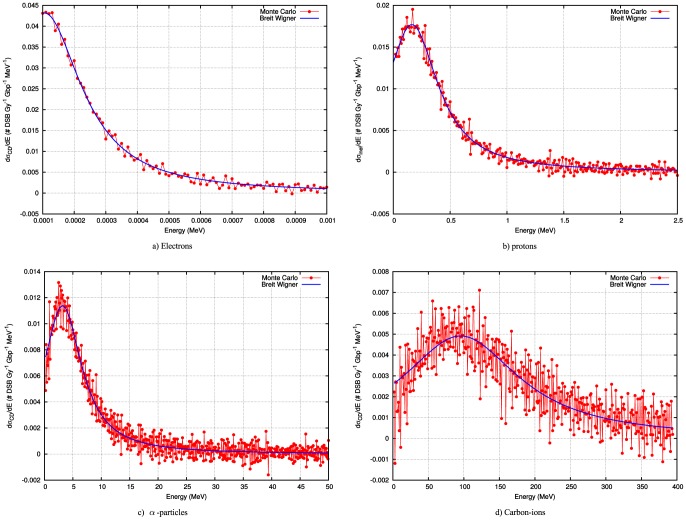
Fitting the Cauchy expression to the energy differential probability of generating DSB's denoted 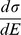.

In Figure 3, the Lorenz expression as outlined in Equation 6 together with a normalization factor, is used to fit the energy differential probability for the generation of DSBs. The fit is performed to minimize the 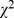
–value. In all cases, the resulting 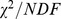 (NDF  =  Number of Degrees of Freedom) are lower than 1. The values of the parameters are provided in Table 1. All fits are completely satisfactory at energies higher than 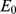. On the lower energy side some discrepancies can be observed depending on the incoming particles, particularly in the case of electrons and carbon ions. We refer the reader to the discussion section. For protons we see a satisfactory fit over the full energy range.

**Table 1:** The different values for 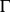 and 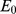 as defined by Equation 9 and obtained from a fitting procedure together with the asymptotic standard error of the fitted parameter.

Particle	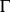	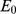	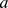	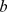
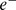	(2.854 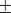 0.051) 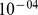 MeV	(1.05736 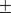 0.036) 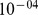 MeV	2.9061	21.460
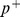	0.5575 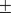 0.0094 MeV	0.1642 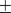 0.0037 MeV	2.89068	21.4273
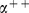	8.20 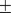 0.17 MeV	3.1850 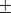 0.056 MeV	3.0856	20.7933
C 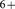	201.7 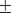 8.4 MeV	95.4 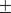 2.5 MeV	3.01459	21.8489

All fits exhibited minimal values of 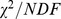 (NDF  =  Number of Degrees of Freedom). The columns 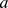 and 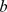 are the parameters indicating the levels of DSB at low, resp. high LET. Note that even in low LET the number of DSB's is not zero as complex damage can occur due to the combination of simple damage events.

**Figure 4:**
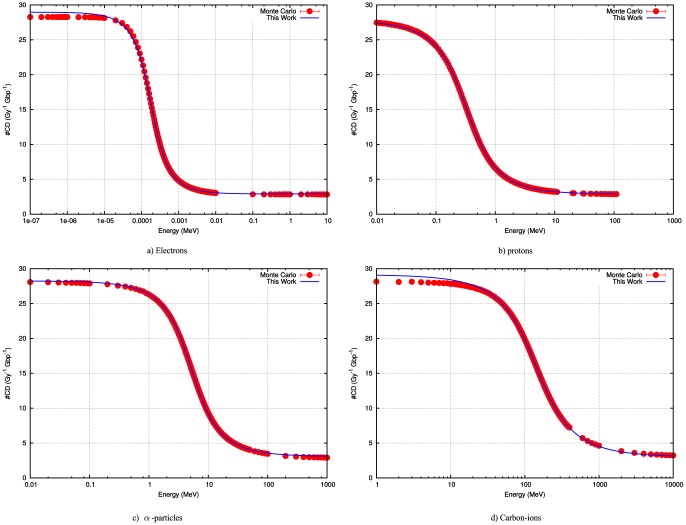
The prediction of the number of double strand breaks or more complex damage as a function of energy for 4 relevant charged particles. This provides the number of Double Strand breaks (DSB) per Gy, Gbp and per cell. The prediction for protons and alpha particles is almost perfect. For electrons and carbon ions some discrepancies exist at lower energies.

Figure 4 shows the final results with all parameters fit. Again, all fits have 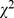
–values commensurate with a positive goodness of fit. The final values and the standard errors for the fitted parameters are listed in Table 1. Note, that the noise in the differential curves increases as the particles become heavier. The random-seeming errors in the estimates of the derivative arise in part from the Monte Carlo estimates of the mean number of DSB per Gy per Gbp and from numerical instabilities associated with the calculation of the derivative using finite difference methods.

### Geometric approach

Now is the time to investigate the geometric interpretation further. To quantify the function 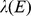 we can use the inelastic mean free path as measure (IMFP). Values for IMFP for electrons are well known in the literature, not in the least as they are important in solid state physics and electron microscopy. They can be found in freely available databases for a variety of elements and compounds, even for organic molecules like DNA [15]. Proton values can be found in a publication by Zhen–Yu and colleagues [16]. For heavier particles such as 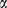
–particles and carbon–ions, the data is more difficult to find. We therefore opt not to use the data for these particles and restrict ourselves to electrons and protons in this further treatment.

In all current microdosimetric codes, the Bethe formalism is used which is valid for higher energies (i.e. above 500eV for electrons). This implies that changes in IMFP, denoted by 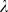, which impact the damage calculated using these codes, also reflect the limitations of the Bethe formalism. From the theory the following expression, with values provided in Table 2, is used:
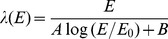
(10)


**Table 2:** Parameters obtained by fitting Eq. 10 to data obtained from NIST (electrons) and Zhen Yu et al. (protons).

Particle	A	B
Electrons	69.200 eV/nm	−153.94 eV/nm
Protons	115.231 keV/nm	−301.45 keV/nm

In this work the parameters 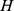 and 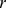 have thus far not been linked to any physical property but were fit. An interesting proposition could be to link these to dimensions of the target structure. Indeed, the choice of a cylinder as a geometric representation is not an accident. It is natural to use the diameter of a DNA–molecule as a measure of the cylinder's diameter. The length of the cylinder is then related to the maximal distance we allow to classify two damage events, being part of the same cluster of complex damage. Both values can readily be found in the literature and text books [17]. For the most prevalent form of cellular DNA (B–DNA), the values are 3.4 nm (i.e. the height of a spiral of 10 base pairs), and 2.37 nm as the diameter. We now define a dimensionless quantity 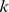 which is specific to the type of charged particle used. It is clear that this parameter acts as a scaling parameter but also depends on the ratio of both fixed parameters. Equation 6 now reads as follows:
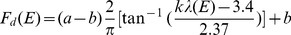
(11)


**Figure 5:**
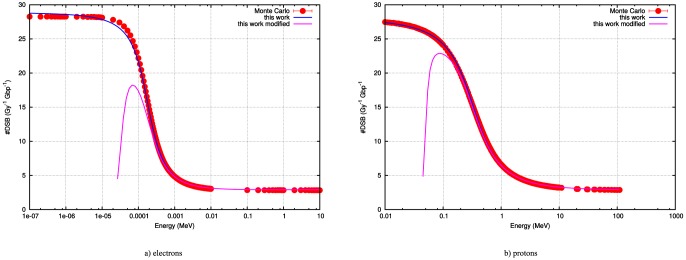
Using the quantities for 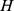 and 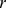, the dimensionless constant 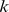 for electrons (left) and protons (right) is determined. Using both the limited expression for 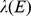 and the more accurate estimate 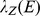. The former provides a fit to the Monte Carlo data comparable with the results obtained using the energy–based formalism. The second approach provides a maximal complex damage yield which differs for electrons and protons.

This reduces the impact of the charged particle's energy on the induction of complex damage in a DNA–molecule to three parameters 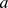, 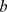, and 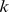. Figure 5 illustrates the use of these parameters and shows that comparable results to the energy–based formalism can be obtained. It follows that we can repeat the fitting procedure keeping 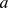 and 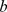 from the expression based on energy (Eq 7). We find values of k = 5.18 for electrons and k = 4.82 for protons.

### Extending the model

In the work presented above as well as in the used Monte Carlo simulations, the Bethe–Barkas formalism together with its flawed approach in the lower energy regions has always been used. It is well established that the IMFP does not follow the expression outlined in Equation 10, where 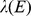 keeps diminishing as the energy diminishes. Indeed, when the energy is lower than 200 eV an increase in IMFP is observed due to plasmonic effects [18]. Ziaja et al [19] showed that it is possible to describe this behavior analytically by extending Equation 10 with a second term as follows:
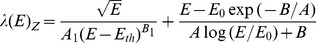
(12)


In this equation the parameter 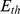 serves as a threshold separating the behavior as described by Bethe from the plasmonic interactions. Using the data provided in the work from Zhen–Yu and colleagues [16] it is straightforward to obtain parameters for the behavior of protons. These are presented in Table 3.

**Table 3:** Parameters as in Table 2 with added lower energy factors.

Particle	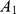	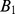	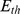	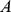	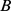	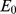
Electrons	0.6560	1.0100	24.2838	65.898	−128.23	1.0
Protons	0.681	1.249	42.38	117.01	−318.7	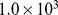

The fitting was performed using Eq. 12.

To extend our model to incorporate the behavior of very low energy particles it is sufficient to replace the expression 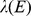 by 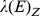 in equation 11. In Figure 5, the modified curves show the difference with the calculations based on the Bethe formalism only. This also shows that there is an upper limit to the increase in DSB's which depends on the type of particle. It is conceivable that this approach also works for the heavier particles which can be seen when using the IFMP's in water for these (not shown).

### Implementation in dose deposition calculations

#### Mono–energetic treatment

In dose calculations a dose matrix is obtained on a dose grid Let 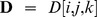 be the dose matrix provided. Then we can write the amount of complex damage incurred by particles with an energy (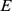) as a damage matrix, denoted as (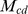). as follows:
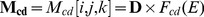
(13)



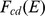
 then denotes a response function converting dose to damage.

#### Poly–energetic treatment

Dose deposition spectra rarely consist of a field of mono–energetic electrons. For a photon source with a given photon spectrum, an energy depositing electron fields exists, which is roughly constant throughout the target volume. Using Monte Carlo simulations it is possible to calculate this field and its spectrum 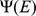. It then becomes possible to include the spectrum in the calculation of the damage matrices. This approach has been used already by different authors [25], [26].
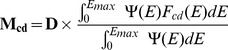
(14)


In the case of charged particle treatment, the particles are moderated and the energy spectrum changes depending on the position of the point where the dose is being deposited. It is therefore necessary to apply Equation 14 to each point separately with knowledge of the depositing energy spectrum in that point. Due to the closed nature of the formalism developed in this paper, it becomes feasible to use off the shelf computing equipment.

#### Application: Proton treatment

Recently, the coupling of Monte Carlo simulations in dose deposition to micro-dosimetric code has been proposed and applied by several groups [25], [26]. Here a two step approach is followed; 1) a general purpose Monte Carlo code (MCNPX 2.7b) [27] is used to estimate the spectrum of all different dose contributing particles, 2) a micro dosimetric code [13] is used to determine the biological damage.

**Figure 6:**
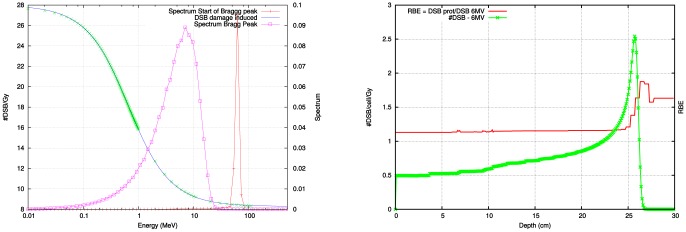
In the left hand figure the spectrum at the beginning of the Bragg peak (scaled by 0.1) is completely within the low LET regimen. While at the end of the Bragg peak a significant part of the dose depositing protons exhibits high LET characteristics. The right hand figure shows the 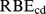 (red line) together with the damage induced by a mono–energetic proton beam.

The framework for conversion of dose to biological effect is implemented on a simulation of a pristine 200 MeV proton beam, taking into account the changing proton spectrum. The proton simulation is performed using MCNPX. Figure 6a shows the variation of the number of complex damage events as a function of energy of the proton. In addition, the spectrum of depositing protons is shown at a position before the Bragg peak and at the Bragg peak. In Figure 6b the effect on the dose deposition is shown together with the 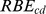 calculated as the complex damage yield generated by the protons at that particular position, divided by the complex damage induced by a 6 MV photon beam with the same spatial characteristics. Note, that the 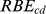 is of the order of 1.1 with larger value of 2 a few mm distal from the Bragg peak. This is commensurate with the cell data reported by Paganetti et al. [28] and Chaudhary et al. [29], who showed that the radiobiological effect at the distal end of a spread out bragg peak increases, a fact predicted by Goitein [30]. Currently, data of direct measurement of DNA–damage in–vitro along a proton beam are scarce. The advent of 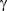
–H2AX measurements, as a marker for DSB–damage is promising in this regard and has been used to investigate anti–protons [31].

## Discussion

We developed an approach to predict damage in complicated situations where fields of different charged particles and their respective energy spectra impact on living cells. The approach, due to its analytical nature allows very fast calculation of damage in otherwise long simulations. In the derivation of this approach using energy alone there are no assumptions on the mechanics with which DNA–damage is caused by the charged particles. The only assumption is that there is a sensitive volume where, if ionizations take place, damage is introduced in the DNA. How exactly this damage is caused is not specified. In the remainder of the text a parameter is identified, the average distance between ionizations for the given charged particle in the medium (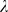). We show that this approach adequately quantifies the results from Monte Carlo simulations based on phenomenological data and reduces these to a closed analytical expression whereby the type of charged particle is expressed by a single parameter (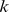). On the other hand we should be aware that issues like repair mechanisms and oxygen effects are not present in the model, making its applicability limited. However, if all things are identical (i.e. the type of cells, oxygenation, etc…) and the only thing different is the type of charged particle and its energy, then the original damage introduced in the DNA structure should correlate with the outcome. An underlying assumption here is that the repair processes are somehow independent from the modality with which the cell is irradiated.

The results of this approach can be applied to determine the biological impact of radiation in mixed environments, as in the case of proton therapy, where protons, electrons and heavier ions (due to neutrons), deposit energy. Other approaches have been proposed to try to predict outcomes from mixed fields, which are based on available clinical response data. Most notably, an approach based on the local effect model (LEM), where macroscopic response data in the form of dose–effect curves is used to quantify the relative effect of the dose delivered [20]. The parameterization, however, of the latter approach is extensive due to the fact that every effect curve has two parameters for a given 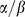
–value, making the model over–parameterized. As such, it is possible to have this model reflect the current knowledge of dose and modality response adequately, which forms an important, albeit controversial tool [21], [22]. Its power to predict the behavior outside of the current knowledge therefore seems to be limited.

Cucinotta et al. attempted to incorporate the volumetric properties of the dose deposition [23] to account for differences in track structure. They observed that: “LET is a poor descriptor of energy deposition in small volumes because of the diffusion of secondary electrons out of the volume and contribution of 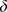
–rays that pass outside of the volume”. To address this problem a quantification of the energy distribution of generated secondary particles, or 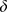
–rays was proposed.

Such a secondary charged particle indirectly changes the behavior with respect to the DNA damage induced. Indeed, depending on the median energy of the spectrum the DNA damage changes accordingly if the dose is kept constant. In the paper presented here this behavior could be easily incorporated by considering the DNA damage for all the particles (i.e. ions and 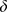
–rays) separately using a methodology modeled on the use of the electronic equilibrium concept in photon cavity theory. Currently this behavior is hidden in the 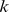 parameter and it would be interesting to see if such an approach will lead to a convergence of all 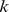
–values for all particles.

To take these actions fully into account an approach to provide a more detailed model of the biological effect directly in the Monte Carlo simulation is proposed by Sato et al. [24] This would, in theory, allow a direct calculation of the effect in terms of energy deposited. However, as outlined by Cucinotta this is not without problems as the behavior of low energy electrons needs to be adequately modelled. This work predicts that the current knowledge using the Bethe formalism, might not be suitably extended.

The results from the geometric interpretation indicate that the overall behavior of the DNA damage induction is identical for all types of charged particles. The only difference is in the dimensionless parameter 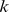. The latter seems to change as the ion used is heavier. Preliminary calculations using the IMFP in water indicate that the value of 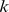 diminishes as the charged particles used are heavier (or more charged, data not shown). A possible reason for this is that the track structure can be quite different for different charged particles. This fact could also be an explanation for the discrepancy found at very low energies for carbon–ions. Indeed, allowing the parameter 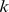 to be covariant with the other parameters, does provide a more adequate fit (data not shown).

The results for the electrons also shows a discrepancy with regard to the generation of complex damage at lower energies. For electrons, the data on very low energy electrons are not available in terms of energy deposition. Indeed, the model proposed here shows a much lower incidence of complex damage due to plasmonic effects in that region.

In summary, the model proposed here allows extension to very low energies for electrons and protons. The fact that there are indications that the induction of DSB's varies linearly with dose, provides an easy implementation to dose planning systems, given the knowledge of dose deposition spectra in a treatment beam. An example of such implementation is provided in the results section.
